# Response to Commentary on “A Population‐Based Correlation Analysis Between Hemoglobin A1c and Hemoglobin Levels”

**DOI:** 10.1111/1753-0407.70086

**Published:** 2025-04-28

**Authors:** Tingyu Zhang, Bin Cui

**Affiliations:** ^1^ Kunming First People's Hospital Kunming Yunnan China; ^2^ Ruijin Hospital Shanghai Jiao Tong University School of Medicine Shanghai China

**Keywords:** estrogen, hemoglobin, hemoglobin A1c

Dear Editor,

We appreciate the author's attention to our study [1] and their detailed comments. To address their questions and help the readers better understand this research, we would like to provide the following explanation of our study's process.

A study titled “Altitudes and Hemoglobin A1c Value” conducted by my colleagues was published in 2024 [2]. Their analysis, which included 95 052 individuals across 162 sites in China, revealed a positive correlation between altitude above 2500 m and HbA1c levels, while no such correlation was observed at altitudes below 2500 m. Due to the lack of data on hemoglobin concentrations and red blood cell counts, their study could not provide more in‐depth results. Fortunately, our team had some data suitable for further investigation. We selected two cities with altitudes below 2500 m: Kunming (1891 m) and Chengdu (503 m). The hemoglobin concentration in Kunming (mean: 158.73 g/L, SD: 16.36) was higher than in Chengdu (mean: 145.44 g/L, SD: 16.82). There was no difference in HbA1c levels between two groups, with Kunming showing a mean of 5.40 (SD: 0.48) and Chengdu 5.41 (SD: 0.41).

Although hemoglobin levels in the two cities differ, they remain within the normal reference range. Moreover, residents of both cities shared similar lifestyles and socio‐economic conditions; we combined the data to analyze the effects of gender and age. We employed the Generalized Additive Model (GAM) for our analysis, as it effectively captures nonlinear relationships and allows us to focus on trends and patterns. Notably, Figure D reveals a significant gender‐based difference in the relationship between HbA1c and age. The HbA1c curve for women shows a distinctive turning point around age 45, which prompted our further investigation in Figures C and D. In Figure D, the disparity in HbA1c levels between women above and below the age of 45 is likely influenced by menopause and changes in estrogen levels. Unfortunately, our dataset does not include estrogen‐related data, preventing further analyses in this direction. However, a previous study indicated that estrogen therapy in postmenopausal women with type 1 or type 2 diabetes can reduce HbA1c and fasting glucose levels, which supports our findings [3].

We acknowledge that our analysis has limitations regarding the types and scope of the real‐world clinical data. Therefore, the findings presented in this article underscore the need for more epidemiological studies with rigorous system design to achieve more reliable conclusions.

## Author Contributions

T.Z. and B.C. drafted and revised the manuscript.

## Conflicts of Interest

The authors declare no conflicts of interest.
